# Postmortem Biochemistry and Immunohistochemistry in Anaphylactic Death Due to Hymenoptera Sting: A Forensic Case Report

**DOI:** 10.3390/ijerph20095640

**Published:** 2023-04-25

**Authors:** Cristina Mondello, Gennaro Baldino, Vincenzo Cianci, Elena Forzese, Alessio Asmundo, Antonio Ieni, Elvira Ventura Spagnolo

**Affiliations:** 1 Department of Biomedical and Dental Sciences and Morphofunctional Imaging, University of Messina, Via Consolare Valeria, 98125 Messina, Italy; 2 Department of Human Pathology of Adult and Childhood “Gaetano Barresi”, University of Messina, Via Consolare Valeria, 98125 Messina, Italy

**Keywords:** anaphylaxis, insect venom, Hymenoptera sting, anaphylactic death, postmortem biochemistry, tryptase, post mortem immunohistochemistry, forensic pathology

## Abstract

Background: Postmortem assessment of anaphylactic death is a challenge for forensic pathologists. One of the most frequent elicitors of anaphylaxis is insect venom. Here, a case of anaphylactic death due to Hymenoptera stings is reported to highlight the contribution of postmortem biochemistry and immunohistochemistry in assessing the cause of death. Case report: A 59-year-old Caucasian man working on his farm was presumably stung by a bee and died. He had a history of previous sensitization to insect venom. The autopsy revealed no signs of insect puncture, mild edema of the larynx, and foamy edema in the bronchial tree and lungs. Routine histology showed endo-alveolar edema and hemorrhage, bronchospasm, and scattered bronchial obstruction due to mucus hyperproduction. Biochemical analysis was performed, and serum tryptase was equal to 189 µg/L, total IgE was 200 kU/L, and specific IgE dosage was positive for bee and yellow jacket species. Immunohistochemistry for tryptase detection was carried out, revealing mast cells and degranulated tryptase expression in the larynx, lungs, spleen, and heart. These findings led to the diagnosis of anaphylactic death due to Hymenoptera stings. Conclusions: The case highlights that the role of biochemistry and immunohistochemistry in the postmortem assessment of anaphylactic reactions should be stressed by forensic practitioners.

## 1. Introduction

Anaphylaxis is commonly known as a “severe, life-threatening generalized or systemic hypersensitivity reaction” and can occur both with immunological and non-immunological mechanisms [1,2].

An anaphylactic reaction is an acute IgE-mediated hypersensitivity response mediated by inflammatory mediators released in systemic circulation from mast cells and basophil; an anaphylactoid reaction has non-IgE mediated mechanisms and, usually, is clinically indistinguishable from an IgE-mediated reaction [3].

The most common triggers of anaphylaxis are drugs, food, and insect venom, and among these, Hymenoptera stings are quite represented [4,5,6,7].

The Hymenoptera order is classified into three families: bees (Apidae), wasps (Vespidae), and ants (Formicidae). These arthropods can sting humans, having the potential to cause anaphylactic and non-anaphylactic reactions. Honeybees and bumblebees have barbed stingers and generally sting only if provoked; they characteristically die after a single sting. Wasps, hornets, and most yellow jackets have no barbed stingers and can sting many times. They are usually more aggressive than bees and also sting without any provocation [8].

Hymenoptera toxins contain various complexes of peptides, enzymes, proteins, and chemicals, and they cause cellular injury via several mechanisms [9]. Studies on the effect of these molecules demonstrated an action similar to toxins, hormones, antibiotics, and defensins which are able to interact with different pharmacological targets, causing inflammation, pain, changes in blood pressure and heart rhythm until cardiac arrhythmia, and neurotoxicity, and are even able to lead to death [10].

It is important to underline that in the evaluation of deaths probably related to a bee sting, it is not always possible to appreciate macroscopic signs of the sting [11]. In fact, in some cases, the sign of the puncture can be difficult to locate, or it is absent. Moreover, if death occurs in a very short time, no local reaction can be found [12].

Therefore, in such cases, the postmortem assessment of the cause of death needs biochemical and immunohistochemical investigations that, together with circumstantial data, clinical data, autopsy, and routine histological findings, can provide useful evidence [13].

Here, the authors report a case of anaphylactic death due to Hymenoptera stings to highlight the contribution of several forensic investigations in assessing the cause of death.

## 2. Case Report

The case regards a 59-year-old Caucasian man with a history of previous sensitization to Hymenoptera sting, the result of which was that, a few years earlier, he had facial edema due to both bee and wasp stings, then confirmed by skin tests. Anamnestic data were negative for cardiovascular and respiratory diseases.

On the day of his death, the man contacted an employee by phone, asking for help and reporting that he was probably stung by bees.

Once arrived, the employee found the man unconscious, lying on the ground, and with a transparent liquid coming out of his mouth, and contacted an ambulance. The medical staff found the man in cardiorespiratory arrest and began resuscitation, but the man died. The autopsy was performed at 24 h after death.

Body inspection showed no signs of bee puncture. The gross examination revealed mild edema of the larynx, whitish foamy liquid in the bronchial tree, and red-brownish foamy dense liquid in the lungs.

**Figure 1:**
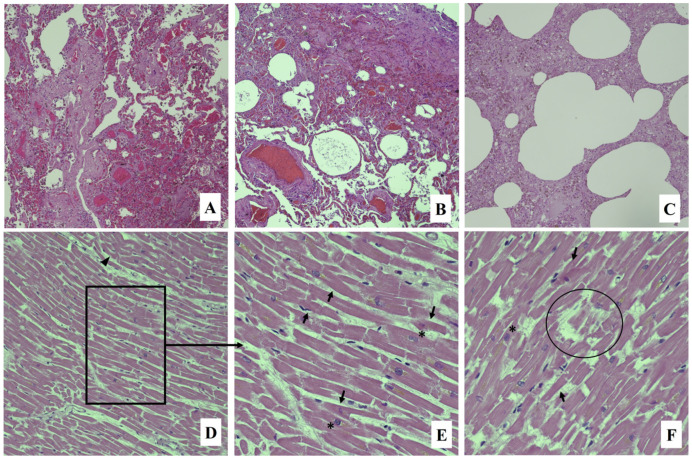
Hematoxylin and eosin staining: lung section with emphysema, endo-alveolar edema, and hemorrhage and marked congestion of the interalveolar septa ((**A**–**C**): 10×); myocardial tissue showing wavy fibers (arrowhead), eosin red areas in myocardial fibers (arrows), fiber breaks, myocyte decay (circle), and square (asterisk) cell nuclei ((**D**): 20×; (**E**,**F**): 40×).

The routine histology was also performed, showing subacute pulmonary emphysema, endo-alveolar edema and hemorrhage, marked congestion of the interalveolar septa, bronchospasm, and scattered bronchial obstruction due to mucus hyperproduction (Figure 1A–C). Myocardial tissue showed hypertrophic myocytes, myofiber break up, and foci of wavy fibers (Figure 1D–E); atherosclerotic plaques were observed in coronary arteries.

The toxicological investigations performed were negative for alcohol, abused substances, and psychotropic drugs.

Biochemical investigations have been performed on the peripheral blood (femoral vein), showing an increased level of tryptase equal to 189 µg/L, troponin I 100,000 pg/mL, and proBNP 579 pg/mL. In addition, the Immuno-CAP method was applied for the determination of total IgE antibodies, which was found to be equal to 200 kU/L. ImmunoCap (Thermo Fisher Scientific/Phadia, Uppsala, Sweden) was carried out for the specific IgE dosage against honey bee (i1), white-faced hornet (i2), common wasp (Yellow Jacket–i3), paper wasp (i4) and yellow hornet (i5); the analysis allowed the identification of honey bee IgE equal to 5.30 kUA/L and yellow jacket IgE of 3.00 kUA/L.

**Figure 2:**
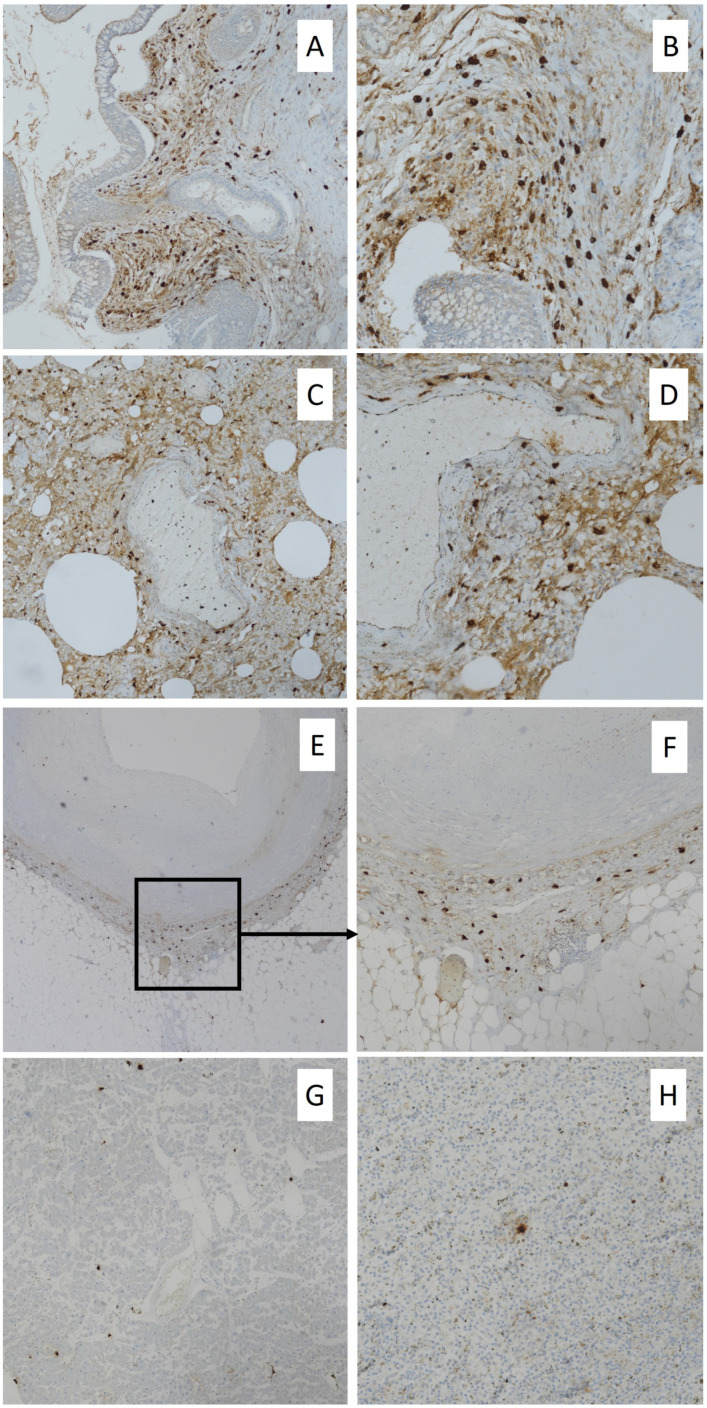
Immunohistochemical findings: mast cells and degranulated tryptase expression in larynx ((**A**): 10×; (**B**): 20×); tryptase expression in lungs with scattered positive mast cells and intense staining of degranulated enzyme ((**C**): 10×; (**D**): 20×); scattered mast cells and degranulated tryptase in coronary wall ((**E**): 4×; (**F**): 10×); mast cell tryptase expression in myocardial tissue with scattered foci of degranulated marker ((**G**): 10×); and spread degranulated tryptase and few positive mast cells in spleen ((**H**): 20×).

For immunohistochemical procedures, 4-micron thick sections obtained from larynx, lung, heart, and spleen tissue blocks were deparaffinized, then washed in descending alcohol scale, treated with 3% hydrogen peroxide for 10 min, washed again in deionized water three times, and incubated with normal sheep serum to prevent unspecific adherence of serum proteins for 30 min at room temperature. After, slides were washed with deionized water and incubated for 30 min at 37 °C with primary anti-human antisera monoclonal mouse anti-tryptase antibody (Roche Diagnostics code 760-4276). Next, the sections were washed three times with PBS and incubated with a biotinylated goat anti-mouse IgG secondary antibody (1:300; Abcam, code ab7064) for 20 min at room temperature, subsequently incubated with horseradish peroxidase-labeled secondary antibody for 30 min, developed with diaminobenzidine tetrahydrochloride, and counterstained with hematoxylin using the ULTRA Staining system (Ventana Medical Systems). Negative controls were obtained by omitting the specific antisera and substituting PBS for the primary antibody. Immunohistochemical reaction revealed intense expression in the larynx and lungs, showing several immunopositive mast cells and spread immunopositivity for degranulated tryptase (Figure 2A–D); mild expression in both coronary arteries’ walls and myocardial tissue characterized by scattered positive mast cells and foci of tryptase degranulation (Figure 2E–G); and mild positivity in splenic tissue showing mast cells and spread degranulated tryptase expression (Figure 2H).

## 3. Discussion

The global incidence of anaphylaxis is reported between 50 and 112 episodes per 100.000 person years with a low mortality rate, estimated at 0.05–0.51 per million people/year for drugs, at 0.03–0.32 for food, and at 0.09–0.13 for venom [14]. In Italy, Bilò et al. [4] reported 392 cases of death from anaphylaxis, with a mortality rate of 0.51 per million people per year. Hymenoptera stings were responsible for 5.6% of these deaths, with an overall mortality rate of 0.17 per million people per year.

Even if Hymenoptera stings are a frequent cause of anaphylactic reactions, there is a consistent number of related deaths that cannot be correctly identified due to the difficulty of making a postmortem diagnosis [15,16].

To perform the diagnosis of death due to anaphylactic shock, it is necessary to integrate circumstantial and anamnestic data, autopsies, and histological findings [17]. However, postmortem assessment of anaphylaxis as the cause of death is considered a challenge for forensic pathologists, because evidence emerging from autopsy and histology is often unspecific. In this context, other postmortem analyses, such as biochemistry and immunohistochemistry, can provide a useful contribution.

The subject’s clinical history serves to collect information on both previous allergic reactions and sensitization phenomena to specific allergens; likewise, the circumstances of death play an important role in the forensic analysis of the case [18].

Relevant findings can be provided from autopsy and routine histology. Sting signs and the evidence emerging from gross and microscopic analysis of the respiratory system (such as laryngeal edema, tracheo-bronchial hypersecretion, bronchoconstriction, emphysema and acute pulmonary edema, congestion, and intra-alveolar hemorrhage) support the occurrence of anaphylaxis [19]. However, some of these findings could not be found and, even if identified, cannot be considered pathognomonic and specific. In fact, such respiratory system involvement has also been described in asthma [18].

Many researchers have suggested the use of biochemistry and immunohistochemistry to fill the gaps related to the poor or absent autopsy and histological data in performing the postmortem diagnosis of anaphylaxis [20].

Particularly, blood biochemical investigations to evaluate tryptase and IgE are described as useful tests to confirm deaths related to anaphylactic reactions due to bee venom, especially when there are no evident signs of stings [21].

Serum tryptase is a neutral protease of human mast cells, mostly used as a biomarker to better define the postmortem diagnosis of anaphylaxis [16,22].

Tryptase is a very stable enzyme and can be detected up to 6 days after death [18]. Nevertheless, it must be emphasized that postmortem degradation processes can cause a reduction in the real concentration of tryptase proportionally to the increase in the postmortem interval (PMI). Therefore, if there is a suspicion of death related to anaphylaxis, it is suggested to collect a blood sample as soon as possible [18].

Forensic literature reports variable cut-offs for serum tryptase from peripheral blood. Meyer et al. [21] demonstrated that a level of tryptase of 10 μg/L or greater has a sensitivity of 86% and specificity of 88% for the diagnosis of postmortem anaphylaxis. Tse et al. [23] reported a cut-off value of tryptase ≥53.8 μg/L on peripheral blood taken from the femoral vessels to make a postmortem diagnosis of anaphylaxis-related death. Edston et al. [24] have proposed a value of 45 μg/L as a new cut-off point, especially if death is due to insect stings.

The literature also offers evidence on serum tryptase measurement on blood taken from central vessels, such as the aorta, in which the suggested cut-off value is 110 µg/L [18]. However, it was highlighted that prolonged cardiac massage or defibrillation can determine the increase in mast cell degranulation and the increase in tryptase levels, due to visceral trauma from chest compressions [25]. In general, several studies suggest preferring peripheric blood sampling for the postmortem tryptase assay [26,27]. Nevertheless, factors affecting the tryptase concentration (i.e., hemolysis, length of agonal period, the specific type of trauma, and cause of death) should always be considered in forensic practice [22]. In fact, the increase in tryptase levels was also described in non-anaphylactic deaths, such as sudden infant death syndrome, acute deaths after heroin injection, traumatic deaths, and asphyxia [16].

The serum concentration of tryptase found in the femoral blood of the case presented here was equal to 189 µg/L and supported the occurrence of anaphylaxis.

In the presented case, other useful data were obtained from the analysis of total and specific IgE. Particularly, the analysis revealed a total IgE value of 200 kU/L and the presence of specific IgE for honey bee (5.30 kUA/L) and yellow jacket species (3.00 kUA/L), demonstrating a high (Radio-Allergo-Sorbent-Test class 4) and moderate (Radio-Allergo-Sorbent-Test class 3) level of sensibilization, respectively.

Evidence in the literature suggests combining results of both mast cell tryptase and allergen-specific IgE and/or total IgE assay in postmortem serum to support the assessment of IgE-mediated fatal anaphylaxis [21,28]. Even if few forensic studies investigated the behavior of postmortem serum total and specific IgE, some evidence showed relative stability of the antibodies in peripheral blood; albeit, some authors reported an increase in total IgE level proportionally with the postmortem interval [25,28,29]. It is also important to observe that the measurement of serum IgE provides information about the atopic disposition and degree of sensitization to a particular allergen; thus, this cannot be considered a confirmation of the causal link between IgE-mediated anaphylaxis and death [25,28].

Immunohistochemistry is another investigation useful for postmortem diagnosis of anaphylaxis. Many studies focused on the role of mast cell and tryptase detection in tissues, among which are bronchial, respiratory, and intestinal mucosa, and the red pulp of the spleen and connective tissue (i.e., cutaneous and perivascular) [18]. Nevertheless, it is important to underline that the identification of mast cells in the tissues cannot be considered sufficient to make a diagnosis of certainty. These limits are related to (i) the involvement of mast cells in various biological processes (i.e., tissue remodeling, angiogenesis, fibrosis, and asphyxia), (ii) the physiological interindividual variability in the number of mast cells, and (iii) the increased detection also observed in non-anaphylactic death [18,30]. Particularly, Edston et al. [30] reported a similar number of pulmonary mast cells in both anaphylactic deaths and control cases (cardiovascular deaths), whereas a higher expression of spleen mast cells was observed in anaphylactic deaths rather than in controls.

The immunohistochemical analysis performed in the presented case revealed an intense positivity of mast cells and degranulated tryptase in the larynx and lungs, together with a mild marker expression in the spleen. These expression patterns are in accordance with the evidence in the literature and support an anaphylactic death. Moreover, the immunohistochemical findings observed in the heart, together with the increased level of serum troponin and pro-BNP, could suggest a coronary hypersensitivity similar to that described in Kounis syndrome. This morbidity is associated with allergic, hypersensitivity, anaphylactic, and anaphylactoid reactions, and it is classified into three types [31].

The type I variant, known as vasospastic allergic angina, is characterized by endothelial dysfunction or microvascular angina and occurs in subjects with normal or nearly normal coronary arteries and in the absence of predisposing factors for coronary artery disease; the releasing of inflammatory mediators due to anaphylaxis can cause coronary artery spasm until myocardial injury with impaired cardiac enzymes and troponins. The type II variant, also known as allergic myocardial infarction, has been described in subjects with quiescent pre-existing atheromatous disease. In this case, the acute release of inflammatory mediators can provoke both coronary artery spasms with normal cardiac enzymes and troponins or coronary artery spasms associated with plaque erosion or rupture. The type III variant occurs in patients with coronary artery stent in whom the inflammatory reactions cause a prothrombotic response and the stent thrombosis; eosinophils and mast cells are generally detected in thrombi and coronary wall at histological examination [31].

Therefore, in Kounis syndrome, the myocardial damage seems related to the effect of both mast-cell degranulation and the release of inflammatory mediators that affect the cardiovascular system (i.e., coronary vasoconstriction induced from histamine) [31]. Few cases of Kounis syndrome due to bee and wasp stings have been described in the literature [32,33].

The case argumentation highlights that postmortem diagnosis of anaphylactic death is based on a combination of data about the event, medical history, gross and microscopic examination, and blood serum analyses. Particularly, even if tryptase analysis by biochemistry and immunohistochemistry and IgE dosage have limits in specificity and sensitivity, their integration with the other information is fundamental to performing a differential diagnosis and, thus, to assessing anaphylaxis [34]. Moreover, a prompt sampling, performed as soon as possible, is crucial to prevent the effect of postmortem phenomena (i.e., cell lysis) on tryptase [35].

## 4. Conclusions

In conclusion, data emerging from the forensic investigations lead to assessing the cause of death as an anaphylactic shock due to Hymenoptera stings affecting the respiratory system and the cardio-circulatory system, with possible vasospastic involvement of coronaries. The case described here supports the importance of circumstantial data in guiding postmortem investigations, especially if no external signs attributable to the insect bite and/or unspecific autopsy and histological evidence are found. Moreover, the important role of biochemistry and immunohistochemistry in demonstrating the anaphylactic reaction has been described, suggesting that these investigations should be routinely implemented in forensic practice when anaphylaxis is suspected.

## Data Availability

All data have been included in the paper.
